# Interventions to improve the review of antibiotic therapy in acute care hospitals: a systematic review and narrative synthesis

**DOI:** 10.1093/jacamr/dlaa065

**Published:** 2020-09-17

**Authors:** Ayodeji Matuluko, Jennifer Macdonald, Valerie Ness, Kay Currie

**Affiliations:** Safeguarding Health through Infection Prevention (SHIP) Research Group, School of Health and Life Sciences, Glasgow Caledonian University, Glasgow, UK

## Abstract

**Objectives:**

To synthesize current evidence for the effectiveness of interventions to ensure the timely review of antibiotics in acute care hospitals.

**Methods:**

Five databases were searched from 1 January 2015 to 8 March 2019 for studies in English, focused on the timely review of antibiotics in acute care hospitals. Randomized controlled trials, non-randomized studies, case–control and cohort study designs were eligible. Intervention strategies were categorized according to the Cochrane Effective Practice and Organisation of Care taxonomy of health interventions, then mapped to the intervention functions of the behaviour change wheel.

**Results:**

Fourteen studies were included. Most studies (11 out of 14) were conducted in single sites. Nine out of 14 reported intervention delivery by more than one healthcare professional. Physicians were the main targets of interventions in all studies. Thirteen out of 14 studies tested interventions comprising more than one strategy. The three most commonly utilized strategies within interventions were clinical practice guidelines, audit and feedback, and educational materials. Only one study employed theory in intervention evaluation. Reported interventions led to timely review and switch of IV antibiotic therapy, and shortened durations of overall antibiotic therapy.

**Conclusions:**

Interventions to improve the review of antibiotics were found to be effective in the short to medium term, with limited evidence of long-term sustainability in multiple sites. Future research may benefit from the application of theory to intervention design and detailed specifications of interventions to aid their easy replication and implementation in different contexts.

## Introduction

Globally, in acute care hospitals, at least one-third of inpatients receive at least one antimicrobial during their admission.^1^^,^^2^ Versporten *et al.*^1^ estimate that overall, greater than two-thirds (89.3%) of these antimicrobials are IV antibiotics. This high use of IV antibiotics impacts on the overall consumption of antimicrobials reported in acute care hospitals, as well as increasing the risk of the development of antimicrobial resistance (AMR) and *Clostridioides difficile* infection (CDI) due to inappropriate or prolonged usage of antimicrobials.^3^ Furthermore, IV therapy is associated with various complications such as phlebitis, line infection, pain, infiltration and extravasation.^4^

In acute care hospitals, IV and oral antibiotics are widely administered for a variety of indications including community-acquired pneumonia (CAP), skin and soft tissue infections, respiratory tract infections, urinary tract infections, bone or joint infections and sepsis.^1^ The initiation of IV antibiotics is commonly the first intervention when patients present to hospital with suspected infections.^1^^,^^2^ It is important to optimize IV antibiotic therapy following initiation, influenced by subsequent changes in patients’ clinical parameters and the results of laboratory investigations for infection.^5^ This optimization of antibiotic therapy is the underlying principle of antimicrobial stewardship (AMS), which is ‘a set of coordinated strategies to improve the use of antimicrobial medications with the goal of enhancing patient health outcomes, reducing resistance to antibiotics, and decreasing unnecessary costs’.^6^

In practising AMS, the ideal subsequent actions following the start of IV antibiotics are to review the IV antibiotic prescription within 24 to 72 h and document evidence of continued need for administration of that antibiotic (through the results of microbiology tests or ongoing clinical symptoms); or change the antibiotic, either to another IV antibiotic with a different spectrum (de-escalation) or to an oral antibiotic [commonly called IV to oral antibiotic switch (IVOST)]; or lastly stop the antibiotic when there is no evidence of infection.^5^^,^^7^

Benefits of early review and switch of IV antibiotics to oral antibiotics include freeing up time that is involved in the preparation and administration of IV antibiotics, shortening patients’ length of stay (LOS) in hospital, reducing the incidence of adverse effects associated with IV antibiotic therapy, including AMR, and lessening morbidity and mortality in hospitals.^5^^,^^8^ There is also evidence that shorter durations of treatment or days of therapy (DOT) for IV and oral antibiotics are as safe and effective as longer DOTs.^9^ Despite these documented benefits, review of IV antibiotic therapy and the use of shorter duration of antibiotics are not implemented effectively in practice.^8^^,^^10^

The antibiotic prescribing pathway^11^ involves an iterative process, from the determination of the presence of an infection, to the initiation of antibiotic therapy according to guidelines, and then review of therapy based on further clinical investigations. These different stages along the antibiotic prescribing pathway^11^ are achieved by the key actions of different categories of healthcare professionals (HCPs), e.g. physicians, nurses, microbiologists and pharmacists. Interventions to improve antibiotic prescribing and promote AMS have been studied extensively.^12^ These interventions, focused on different aspects of the antibiotic prescribing pathway,^11^ have been shown to be clinically effective for hospital inpatients in an updated Cochrane review.^12^ Reported interventions led to increased appropriateness of antibiotic therapy, reduced antibiotic consumption, reduced duration of antibiotic therapy, reduced lengths of hospital stay and no likely increases in mortality.^12^ However, as the review authors highlighted, there is a lack of theory application to the design of AMS interventions and no sufficient investigation of the contexts within which they are applied.^12^

### Rationale

In light of the existing evidence and recommendations from the Cochrane review by Davey *et al.*,^12^ this systematic review was carried out to determine whether research conducted on interventions to improve the review of antibiotic therapy (and subsequent actions, i.e. IVOST, stop, de-escalate)—since the Cochrane review was published—have addressed the key gaps identified by the authors. In particular, this review explores whether there has been an improvement in the design of AMS interventions, i.e. with a theoretical underpinning.

### Objectives

This systematic review was carried out with the main objective of identifying and describing the current evidence base of the effectiveness of interventions—in acute care hospitals—which have been utilized to ensure the (i) timely review of IV antibiotics; and subsequently (ii) timely IVOST; and (iii) the optimization of the duration of oral and IV antibiotics.

The following are the specific review questions: (i) Which groups of HCPs have been targeted by interventions to improve the review of antibiotic therapy in hospitals? (ii) Which intervention strategies have been employed to improve the review of antibiotic therapy in acute care hospitals? (iii) Are intervention strategies to improve the review of antibiotic therapy effective? (iv) Do interventions to improve the review of antibiotic therapy have a theoretical underpinning, and what are the associations between use of theory in intervention design and effectiveness?

## Methods

This systematic review was conducted according to PRISMA guidelines.^13^

### Protocol and registration

The review protocol was registered with the PROSPERO International Prospective Register of Systematic Reviews with registration number: CRD42019125473.^14^

### Eligibility criteria

The PICO tool^15^ (population/participants/problem, intervention/exposure, comparison/context and outcome) was adapted to ‘PIC^2^OS’ (participants, intervention, comparison, context, outcomes and study design) to develop the eligibility criteria for this review^16^—see Table 1. In addition, only papers published in English (due to lack of translation facilities) and after 1 January 2015 (the last month when a search was carried out in the Davey *et al.*^12^ review) were included.

### Information sources and search strategy

MEDLINE, CINAHL, Embase, Web of Science and PsycINFO databases were searched from 1 January 2015 to 8 March 2019. Four key authors’ profiles on Google Scholar were also searched for any articles not found in the databases. These authors were selected based on their published research around AMS and antimicrobial prescribing in hospitals as identified through the recurrent appearance of their names in published systematic reviews and during database searching. Grey literature searches were carried out in the WHO Library database, key organization websites and conference proceedings (ESCMID, Society for Healthcare Epidemiology of America, Healthcare Infection Society and Infection Prevention Society) and the British Library (Ethos) Collection of PhD dissertations/theses, to identify relevant studies potentially missed during the database search. Reference lists of final selected articles were also searched to retrieve relevant articles.

### Search

Table 2 outlines the different search terms that were used to carry out database searches. A first search was completed on MEDLINE (search strategy reported in Table S1, available as Supplementary data at *JAC-AMR* Online), using a comprehensive list of search terms, and this search was then amended or modified in the subsequent databases depending on the subject headings and keywords and their synonyms identified in the databases. A combination of keywords (searching the title and abstract) and index terms, as well as their synonyms where applicable, were used depending on the database. Spelling variations for different search terms were also employed.

**Table 2.** Search terms^a^

Participants/population	Intervention	Context or setting
healthcare professional*/health care professional* doctor* physician* pharmacy/pharmacist* nurse* clinician*	Core search terms:	hospital* ‘acute care’/‘acute-care’ inpatient*
	antimicrobial stewardship/antibiotic stewardship	
	antimicrobial prescribing/antibiotic prescribing	
	Proximity search terms:	
	intervention*	
	guideline*	
	policy/policies	
	implement*	
	‘audit and feedback’	
	program*	
	‘quality improvement’	

The asterisk (*) stands for truncation, used to search for alternative endings of search terms; ‘’ indicate where terms were searched as ‘one word’ to avoid unlinking of the terms in databases; and ‘AND’ and ‘OR’ Boolean operators were used to combine search terms (see example for MEDLINE in Table S1).

Proximity tools such as ‘N5’, and ‘near5’ were used to retrieve any records where the search terms were within five words of each other (see example for MEDLINE in Table S1).

a An updated search (to the Cochrane review by Davey *et al.*^12^) was carried out initially as a systematic scoping review (A. Matuluko, J. MacDonald, V. Ness and K. Currie, unpublished data) of all interventions to improve AMS published since 1 January 2015 using broad search terms (Davey *et al.*^12^ conducted searches up to January 2015).

### Study selection

Broad screening of all identified titles and abstracts against the eligibility criteria was carried out by the first reviewer (A.M.) to identify potentially eligible studies. A 1% (*n *=* *75) sample of the studies identified at the broad screening stage were checked by a second reviewer (J.M.) to confirm their eligibility. A 97% agreement by both first and second reviewer was achieved. Second-stage (narrow) screening of the full text of relevant papers against the eligibility criteria was conducted by the first reviewer (A.M.). A second reviewer (V.N.) double-checked a 10% (*n *=* *27) sample of all the selected full-text articles against the eligibility criteria. Disagreements were resolved by discussion.

### Data collection process

Key details of included studies were extracted using a structured review-specific data extraction form (Table S2). Data were extracted by one reviewer (A.M.) and checked by a second reviewer (J.M.) for 20% (*n *=* *3) of studies.

### Data items

The following data were extracted: article characteristics (author, year and location/country of origin); study design; study aim/objective; study site(s) (single or multiple); study time period; methods; sample size; descriptions of interventions to improve the review of antibiotic therapy and comparators, if any; interventionists (who delivered the intervention); HCPs targeted by the intervention; theory/theoretical model/framework employed; outcome measures and timepoints; data analysis; and key findings.

#### Types of interventions

To ensure a clear categorization of interventions and also aid potential comparison with future systematic reviews, the individual strategies employed within interventions in this review were categorized using the Effective Practice and Organisation of Care (EPOC) taxonomy of health interventions^17^ and then mapped to the intervention functions of the behaviour change wheel (BCW).^18^ The definitions of the EPOC subcategories and BCW intervention functions are outlined in Table S3(a and b).

The EPOC taxonomy ensures clear categorization of individual strategies employed within broad interventions targeted at healthcare workers, while the intervention functions of the BCW allow classification of the intervention strategies into ‘higher-level’ activities designed to change behaviour (in this case, review of antibiotic therapy). Coding of intervention descriptions to the EPOC taxonomy and BCW intervention functions were carried out by A.M. In addition, all studies were independently coded by J.M. and V.N. (50% by J.M., 50% by V.N.), using the same tools. A few discrepancies in the coding of interventions were recorded. These discrepancies were resolved via discussion and all three reviewers arrived at a consensus on the coding categories.

### Quality appraisal and risk-of-bias assessment in individual studies

Three tools, selected based on the design of each study, were used for quality appraisal and risk-of-bias assessment. The National Heart, Lung and Blood Institute quality assessment tools^19^ were used to appraise the quality of before–after (pre–post) studies with no control group, and observational cohort studies. The Joanna Briggs Institute Critical Appraisal Checklist^20^ was used to appraise the quality of quasi-experimental/non-randomized experimental studies. These studies were labelled either ‘good’, ‘fair’ or ‘poor’ at the stage of quality appraisal. For randomized controlled trials (RCTs) and controlled before–after (CBA) studies, risk of bias was assessed using the Cochrane EPOC-suggested ‘risk of bias’ criteria.^21^

Quality appraisal and risk-of-bias assessment were carried out by the first reviewer (A.M.). A second reviewer (V.N.) independently assessed the quality of three studies, rating the quality of a minimum of one study per tool. Minor discrepancies in risk-of-bias assessment/quality appraisal were recorded, indicating rigour in assessment; these minor discrepancies were resolved via discussion. Studies were not excluded based on their quality because all included studies met the objectives of the review.

### Synthesis of results

A narrative synthesis was conducted due to the heterogeneity of the studies that were included; study design, intervention comparisons and outcome measures particularly varied in the studies. Narrative synthesis involves using text and words to summarize the findings of included studies.^22^

## Results

### Study selection

**Figure 1.**
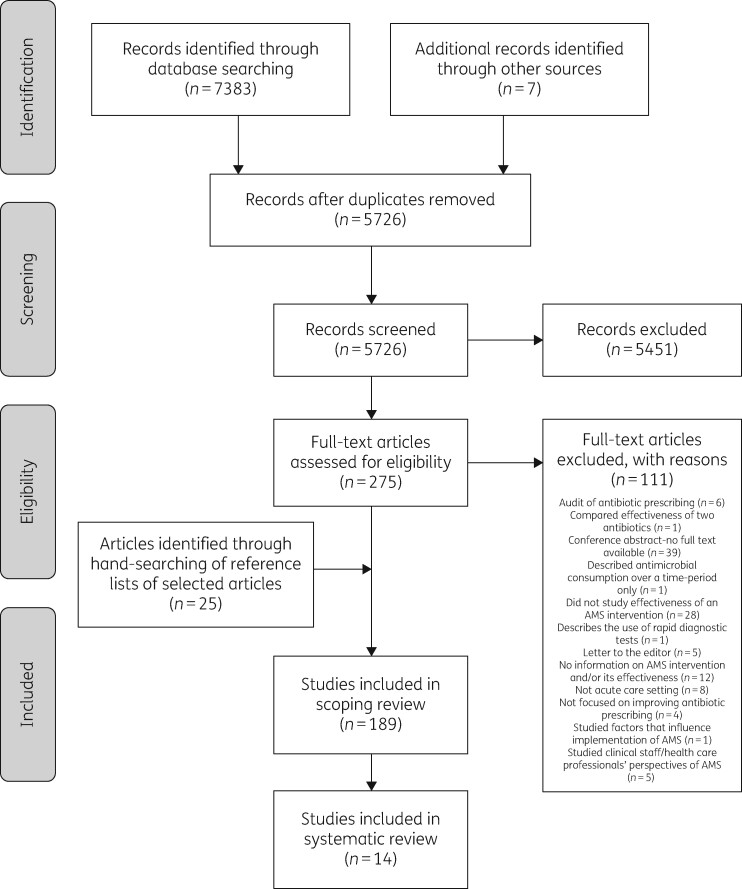
PRISMA^13^ flow diagram of study selection.

A total of 7390 records were identified. After removal of duplicates, 5726 references remained. Broad screening narrowed this to 275 articles, which were screened against the eligibility criteria. The initial scoping review (see footnote below Table 2) identified 189 eligible studies that evaluated various interventions to improve AMS. A final 14 studies, focused on the review of antibiotic therapy, were included in this systematic review.^23–36^ Figure 1 summarizes the study selection process.

### Study characteristics

Table S2 provides an overview of the 14 included studies.

The majority (93%, *n *=* *13) of the studies were from high-income countries (HICs):^23–34^^,^^36^ UK (*n *=* *2), USA (*n *=* *2), Singapore (*n *=* *2), Canada (*n *=* *1), France (*n *=* *1), Ireland (*n *=* *1), Japan (*n *=* *1), Korea (*n *=* *1), the Netherlands (*n *=* *1) and Switzerland (*n *=* *1). The remaining study^35^ was from Malaysia, an upper-middle-income country. Eleven studies were conducted in single sites, i.e. one hospital.^23–25^^,^^27^^,^^29–34^^,^^36^ Of the three remaining studies conducted in multiple sites, Foolad *et al.*^26^ evaluated interventions across three medical centres, Lesprit *et al.*^28^ conducted their study in four university hospitals and Sze and Kong’s^35^ study spanned eight district hospitals.

The studies’ time periods, which included pre-intervention, intervention and post-intervention periods, ranged from as short as 4–7 weeks^27^^,^^33^^,^^36^ to 4–6 months,^25^^,^^32^^,^^35^ 1–2 years,^23^^,^^24^^,^^26^^,^^28^^,^^29^^,^^31^^,^^34^ and 5 years.^30^ Only Sze and Kong,^35^ Eljaaly *et al.*,^25^ Rizan *et al.*^34^ and Berrevoets *et al.*^24^ identified distinct post-intervention follow-up periods (2 months, 3 months, 5 months and 13 months respectively). Also, the study by Loo *et al.*^30^ was the only one to evaluate intervention effectiveness retrospectively over a 5 year period. A broad range of outcome measures—both outcomes of antibiotic therapy review and clinical outcomes—were reported across all studies and are detailed in Table S2.

#### Participants

Physicians were the targets of the interventions in all studies, except in the studies by Foolad *et al.*^26^ and Hobday *et al.*^27^ where pharmacists were also targeted by the intervention(s) in addition to physicians.

#### Interventions

Table 3 outlines the EPOC subcategories^17^ and BCW intervention functions^18^ of the interventions. Out of the 14 studies, 13 studies^23–28^^,^^30–36^ tested interventions comprising more than one strategy. Each individual strategy was identified by coding reported interventions to the EPOC taxonomy subcategories^17^ and these are outlined in the following subsections.

**Table 3.** Intervention subcategories/individual strategies and BCW intervention functions employed in the studies

Study	EPOC taxonomy subcategories^17^										Total per study	Intervention functions of the BCW^18^						Total per study
	audit and feedback	clinical incident reporting	clinical practice guidelines	continuous quality improvement	educational materials	educational meetings	educational outreach visits or academic detailing	local consensus process	local opinion leaders	reminders		education	persuasion	coercion	enablement	environmental restructuring	restriction	
Beeler *et al.*^23^			✔							✔	2	✔			✔	✔		3
Berrevoets *et al.*^24^	✔		✔		✔	✔				✔	5	✔	✔		✔	✔		4
Eljaaly *et al.*^25^							✔	✔			2		✔			✔	✔	3
Foolad *et al.*^26^	✔		✔		✔	✔					4	✔	✔		✔	✔		4
Hobday *et al.*^27^	✔	✔		✔	✔	✔	✔		✔	✔	8	✔	✔	✔	✔	✔		5
Lesprit *et al.*^28^			✔		✔		✔				3	✔	✔		✔			3
Liew *et al.*^29^							✔				1		✔			✔		2
Loo *et al.*^30^	✔		✔								2		✔		✔	✔		3
Niwa *et al.*^31^	✔								✔		2		✔		✔	✔		3
Park *et al.*^32^							✔		✔		2		✔			✔		2
Riain *et al.*^33^			✔				✔				2		✔			✔		2
Rizan *et al.*^34^	✔		✔		✔	✔		✔		✔	6	✔	✔		✔	✔		4
Sze and Kong^35^			✔		✔					✔	3	✔			✔	✔		3
Thompson *et al.*^36^						✔		✔			2	✔			✔	✔		3
Total across studies	6	1	8	1	6	5	6	3	3	5		8	11	1	10	13	1	

Table S2 provides more details on the interventions in each study, as well as the individual strategies contained within them, based on the subcategories of the EPOC taxonomy^17^ and the intervention functions of the BCW^18^ they map to.

##### Targeting the timely review of antibiotic therapy

The time period targeted for the review of antibiotic therapy using various intervention strategies differed in the studies included. Review of antibiotic therapy within 24 h was the focus of intervention strategies in studies by Niwa *et al.*^31^ and Park *et al*.^32^ Lesprit *et al.*^28^ also studied the influence of their intervention strategy on review at Day 1 (24 h). Review within 48 h was the focus of intervention strategies in four studies.^27^^,^^29^^,^^34^^,^^35^ Furthermore, intervention strategies were focused on review within 48–72 h in two studies,^33^^,^^36^ while five studies employed intervention strategies to ensure review at ≥72 h.^24–26^^,^^28^^,^^32^ In addition to reviews at Days 1 and 3, Lesprit *et al.*^28^ focused their intervention strategy on ensuring review at Day 4 (96 h), while Beeler *et al.*^23^ targeted improving review within 60–300 h.

##### Intervention strategies

Based on the EPOC taxonomy, the number of strategies in the interventions within each study ranged from one to eight, with a median of two. Across all studies, ‘clinical practice guidelines’ was the most commonly employed strategy, i.e. either through the development or the promotion of the implementation of clinical guidelines for antibiotic prescribing (*n *=* *8).^23^^,^^24^^,^^26^^,^^28^^,^^30^^,^^33–35^ This was followed by ‘audit and feedback’ (*n *=* *6);^24^^,^^26^^,^^27^^,^^30^^,^^31^^,^^34^ distribution of ‘educational materials’ (*n *=* *6)^24^, 26–28^,34,35^ such as pocket cards containing clinical guidelines, protocols and/or making them available on hospital intranet sites; and ‘educational outreach visits or academic detailing’, which was achieved by review of antibiotic therapy, followed by either verbal or written recommendations for change in therapy or IVOST (*n *=* *6).^25^^,^^27–29^^,^^31^^,^^33^ ‘Educational meetings’^24^^,^^26^^,^^27^^,^^35^^,^^36^ in the form of sessions delivered to different categories of HCPs and ‘reminders’ through posters, prompts, pocket cards, stickers or computerized alerts were each employed in five studies.^23^^,^^24^^,^^27^^,^^34^^,^^35^ ‘Local consensus processes’ were used to promote the implementation of guidelines or agree on the refinement of interventions in three studies.^25^^,^^34^^,^^36^ ‘Local opinion leaders’ were also identified and used to achieve appropriate review of therapy and/or IVOST in three studies.^27^^,^^31^^,^^32^ Hobday *et al.*^27^ employed ‘continuous quality improvement’ and ‘clinical incident reporting’ as part of their suite of interventions.

##### Intervention functions

Six intervention functions of the BCW^18^ were found in the studies, with the number of intervention functions ranging from two to five in each study with a median of three intervention functions per study. The most commonly applied intervention functions across all studies were ‘environmental restructuring’, i.e. changing the physical or social context (*n *=* *13);^23–27^^,^^29–36^ ‘persuasion’, i.e. using communication to induce positive or negative feelings or stimulate action (*n *=* *11);^24–34^‘enablement’ (*n *=* *10),^23^^,^^24^^,^^26–28^^,^^30^^,^^31^^,^^34–36^ i.e. increasing means/reducing barriers to increase capability or opportunity; and ‘education’, i.e. increasing knowledge or understanding (*n *=* *8).^23^^,^^24^^,^^26–28^^,^^34–36^ Only Eljaaly *et al.*^25^ had ‘restriction’ (using rules to reduce the opportunity to engage in the target behaviour) as an intervention function. This intervention involved requiring initial authorization plus re-authorization from an infectious disease (ID) team, for the prescription of certain antimicrobials classified as restricted antimicrobials in the hospital under study, if these antimicrobials were administered for ≥3 days. Lastly, the ‘clinical incident reporting’ strategy in the study by Hobday *et al.*^27^ involved ‘coercion’ (creating expectation of punishment or cost) due to the requirement for pharmacists to fill in an incident report if antibiotic review within 48 h did not take place.

##### Interventionists

Nine out of the 14 studies involved the delivery of the intervention by more than one HCP or a multidisciplinary AMS team that included either a physician or ID specialist physician or pharmacist; a microbiologist; and/or clinical pharmacists.^24–27^^,^^29–33^ Lesprit *et al.*^28^ reported that only AMS ID physicians were responsible for the delivery of the intervention, while only pharmacists delivered the intervention in the studies by Sze and Kong^35^ and Thompson *et al*.^36^ Beeler *et al.*^23^ and Rizan *et al.*^34^ did not mention who delivered the intervention(s) in their studies.

##### Theoretical underpinning of interventions

Only one of the studies^34^ highlighted the use of theory in the evaluation of the effectiveness of a set of intervention strategies targeted at improving concordance with IVOST guidelines and the uptake of these interventions by surgeons. In this study, Rizan *et al.*^34^ used the ‘Roger’s diffusion of innovation’ model^37^ to group general surgeons into five categories and describe intervention uptake across these categories. The Roger’s diffusion of innovation model states that there are five categories of intervention adopters: innovators, early adopters, early majority, late majority and laggards.^37^ Although a theory was employed in this study,^34^ it was not employed in intervention design or in tailoring the intervention to the different categories of adopters.

### Methodological quality and risk of bias within included studies

The before–after and cohort study designs in 12 out of the 14 studies^24–27^^,^^29–36^ allowed for comparisons to be made between the studied intervention(s) and either a control group, usual care, pre-intervention outcomes, or subgroups of samples that received the intervention(s). This meant that it was easier to evaluate the impact of the intervention(s). There was only one RCT, by Lesprit *et al.*,^28^ in which most of the criteria of the risk-of-bias assessment were judged to be of a low risk. The strengths of this RCT^28^ included 1:1 randomization of groups that received either the intervention or usual care, with a clear sample size justification and similar baseline characteristics between both groups of patients. The major weaknesses in this RCT^28^ included the non-measurement of outcome measures before the introduction of the intervention and the risk that the control group may have been exposed to the intervention. The CBA study by Beeler *et al.*^23^ was judged to be of high risk for three criteria of the risk-of-bias assessment and low risk for two criteria, with an unclear risk highlighted for the other three criteria. The major methodological issues in this CBA study^23^ were around the non-random method of allocating study participants to the control and the intervention group and key differences found in the baseline characteristics of both study groups.

Common strengths across all studies include that the study objectives were clearly stated in 13 studies^23–32^^,^^34–36^ and 11 out of the 14 studies^23–29^^,^^31^^,^^32^^,^^34^^,^^35^ clearly specified the study population. However, sample size justification or power description was only provided by Eljaaly *et al.*,^25^ Rizan *et al.*^34^ and Sze and Kong,^35^ which means that they took into consideration the minimum sample size that would be sufficient to observe the intended effect of the intervention. With only three studies^26^^,^^28^^,^^35^ conducted in multiple sites (three, four and eight hospitals in each study), this means that there is limited evidence of the application of interventions across potentially differing settings, thus limiting intervention generalizability across different acute care hospital settings.

As AMS interventions are usually introduced into existing work processes, to even observe an intended change in prescribing practice or the intended outcomes, there needs to be a sufficient period for implementation and evaluation of these interventions. This is lacking in the majority of studies in this review, with insufficient intervention periods allowed to effectively study the effectiveness of the interventions in the long term (i.e. >12 months post-intervention^38^). Only Berrevoets *et al.*^24^ (who included a 13 month post-intervention period) and Loo *et al.*^30^ (who retrospectively evaluated the impact of their interventions over 5 years) gave consideration to the long-term effectiveness of their interventions.

Detailed results of the quality appraisal/risk-of-bias assessment for each study are presented in Table S4(a–d)*.* The quality of each study was not judged to have an impact on the strength of the resultant evidence. However, conclusions drawn from the current evidence took into consideration that studies at higher risk of bias, or which were of lower quality, would limit confidence in the review findings.

### Results of individual studies

Intervention effectiveness was determined by the achievement of the study outcomes as a result of the intervention. For studies where there was a comparison between one or more intervention group(s) and a control group, if there was a difference in study outcomes in favour of any of the intervention groups then the intervention was deemed to be effective. Reports of statistical significance (*P* values) were also used to determine intervention effectiveness, where reported.

#### Intervention effectiveness

Table S5 outlines the detailed findings on intervention effectiveness according to the measured outcomes for each study. These findings on intervention effectiveness are presented in the following subsections, for each outcome measure.

##### Effectiveness of intervention strategies in ensuring timely review of antibiotic prescriptions

Timely review of IV antibiotics was assessed in three studies,^27^^,^^31^^,^^34^ with all three showing improvement in the review of antibiotic therapy.

In the quality improvement project reported by Hobday *et al.*,^27^ the intervention strategies led to: increased completion of the 48 h antimicrobial review box from 68% to 100%; signing of reviews from 66.7% to 100%; and dating of the reviews from 63.3% to 100%, by the 11th measurement in the plan-do-study-act (PDSA) cycle.

In the study by Niwa *et al.*,^31^ daily review (within 24 h) of antimicrobial therapy significantly shortened the number of days to the administration of effective IV therapy, from the onset of infection (*P *=* *0.022).

Lastly, Rizan *et al.*^34^ reported a significant increase in the percentage of patients for whom there was a documented intention to review their IV antibiotics at 48 h post-intervention (*P *<* *0.05). However, they noted that the physicians in this study did not utilize the IVOST prompt sheet to guide de-escalation of IV antibiotic therapy, but how this non-uptake was determined was not reported by the authors.

###### Appropriateness of antibiotic therapy according to guidelines (as a result of timely review)

Lesprit *et al.*^28^ reported a significant increase in the appropriateness of therapy as a result of ID physician review on Days 3 and 4 of therapy, although on Day 1 (when the intervention was also carried out) there was no significant difference in the appropriateness of therapy between the intervention and control groups.

A significant increase (*P *=* *0.001) in the rate of choice of antimicrobials appropriate to the identified pathogens, on the second day from the onset of infection, was also recorded as a result of the intervention by Niwa *et al*.^31^

##### Effectiveness of intervention strategies in ensuring switch from IV to oral antibiotic therapy

Four studies^24^^,^^31^^,^^34^^,^^35^ assessed the rate of IVOST. The intervention strategies in two out of four studies^31^^,^^35^ led to reported significant increases in the rate of IVOST, in favour of the intervention groups.

Intervention strategies by Rizan *et al.*^34^ showed no significant difference in the percentage of IVOST at 48 h, although there was a significant increase in IVOST between 48 and 72 h.

The intervention strategies by Berrevoets *et al.*^24^ were not effective at ensuring IVOST. Berrevoets *et al.*^24^ reported a much more significant reduction in the percentage of IV antibiotic prescriptions of >72 h in the intervention group versus the control group (19.3% versus 6.1%; *P *<* *0.001 and *P *<* *0.05 respectively) in the pre-intervention period. They report, however, that in the post-intervention period a non-significant increase in the percentage of IV therapy for >72 h was observed in the intervention group (*P *=* *0.43), while there was a non-significant decrease in the control group (*P *=* *0.46).

##### Effectiveness of intervention strategies in ensuring reduction in/appropriateness of overall duration of antibiotic therapy

DOT was an outcome measure in 12 studies.^23–30^^,^^32^^,^^33^^,^^35^^,^^36^ DOT was measured in different subgroups of samples in each study, in comparison with either usual care, pre-intervention outcomes or comparison between groups where the physicians targeted by the intervention accepted or did not accept the intervention.

Ten out of 12 of these studies reported significant reductions in DOT or increased appropriateness (according to guideline recommendations) of DOT (*P *<* *0.05) in favour of the intervention strategies.^23–26^^,^^28–30^^,^^32^^,^^33^^,^^35^ Although there were no *P* values of statistical significance reported, Hobday *et al.*^27^ and Thompson *et al.*^36^ reported effective intervention strategies. The suite of intervention strategies by Hobday *et al.*^27^ led to a reduction in the average time on IV antibiotics from a baseline median measurement of 2.25 days to 1.5 days by PDSA cycles 13–14. Thompson *et al.*^36^ reported that the average length of time spent on IV antibiotics was 42% longer (no range quoted) in patients where the physician did not comply with the intervention.

##### Impact of intervention strategies on other clinical/related outcomes

Ten studies reported on the impact of intervention strategies on clinical outcomes such as LOS, mortality, incidence of CDI, and readmission rates to hospital.^25^^,^^26^^,^^28–35^ There was an improvement in LOS in favour of the intervention in four^25^^,^^30^^,^^32^^,^^35^ of six studies that reported on LOS, while there was no difference in LOS in the remaining two studies.^28^^,^^29^ Seven^25^^,^^26^^,^^28^^,^^29^^,^^31^^,^^33^^,^^34^ out of the 10 studies reported on the influence of intervention strategies on mortality, with three studies^25^^,^^29^^,^^31^ noting reduction in mortality rates in favour of the intervention, while the remaining four studies did not record similar improvements. Incidence rates of CDI were measured in three studies,^25^^,^^26^^,^^29^ with no positive changes (as a result of intervention strategies) noted in any of these studies. There were no changes in readmission rates to hospital following discharge in two studies^26^^,^^29^ where this outcome was reported. Antibiotic cost savings, as a result of the intervention strategies, were reported by Loo *et al.*,^30^ Park *et al.*^32^ and Sze and Kong.^35^

Table S5 contains additional details on the influence of intervention strategies on reported clinical outcomes in these studies.

##### Associations between type of intervention strategy and intervention function, and intervention effectiveness

Due to the heterogeneity of intervention strategies and intervention functions, as well as measured outcomes, it was not possible to carry out statistical analysis to determine any associations between types of individual intervention strategies or intervention function, and their effectiveness. However, it is clear from the reported strategies in all studies that improvements in the review of antibiotic therapy can be achieved through the combination of more than one strategy within a set of interventions, as all studies except the study by Liew *et al.*^29^ had at least two strategies within their intervention. A similar trend was noticed with the intervention functions, as each of the intervention strategies in all studies mapped to at least two intervention functions.

The three studies by Berrevoets *et al.*,^24^ Lesprit *et al.*^28^ and Rizan *et al.*,^34^ in which some aspects of intervention ineffectiveness were reported, contained strategies that were found to be effective in other studies, as seen in Table 3. Hence, it is not possible to ascertain which intervention strategies may have contributed to the observed aspects of ineffectiveness in these three studies.^24^^,^^28^^,^^34^

##### Associations between theoretical underpinning and intervention effectiveness

The use of theory in the design of interventions was not evident in any of the studies included in the review. Hence it is not possible to make associations between the use of theory in intervention design and intervention effectiveness, based on the findings of this review.

## Discussion

### Summary of evidence

This systematic review shows that interventions to improve the review of antibiotic therapy are mostly multifaceted, with 13 out of 14 studies^23–28^^,^^30–36^ evaluating interventions that included more than one strategy. In addition, all intervention strategies in the 14 studies mapped to at least two intervention functions. These interventions have been reported to lead to significant improvements in the timely review of antibiotic therapy, in switching from IV to oral antibiotic therapy, and significant impacts on the reduction of the duration of antibiotic therapy. The overall quality of the studies included in this review, and thus the resultant strength of the evidence, is limited by the study designs and the largely uncontrolled nature of the studies (except for one RCT and one CBA study), which generally employed before–after/cohort designs relying on comparisons between a pre-intervention group and a post-intervention group, or two groups of patients that in some cases differed in important baseline characteristics. Hence it is not possible to make concrete claims of causality, i.e. associations between intervention strategy and effectiveness, based on the findings of this review alone.

### HCPs’ roles in intervention delivery

The role of HCPs in intervention delivery is important as different HCPs are involved in the different aspects of the antibiotic prescribing pathway,^11^ which although a non-linear process involves: the prescribing of antibiotics (by either a physician or nurse^39–41^ or pharmacist^39^); the administration of antibiotics (commonly by nurses); their subsequent review and switch from IV to oral antibiotics or de-escalation; and eventual discontinuation of antibiotics. Multi-professional team effort is evident from the studies in this review as 9 out of 14 studies^24–27^^,^^29–33^ included interventions delivered by more than one HCP or a multidisciplinary AMS team. Sole delivery of interventions by a pharmacist also featured in two studies,^35^^,^^36^ reflecting the important role of pharmacists as medicines experts and leads in the delivery of AMS.^42^^,^^43^ However, the specific role and contribution of nurses is not highlighted in these studies. The increasing role of nurses in AMS, especially with developments in nurse prescribing,^39–41^ needs to be taken into important consideration when considering the delivery of AMS interventions. Nurses are important actors in the antibiotic prescribing pathway^11^ and have the potential to impact on the success of AMS interventions.^44^

### The influence of context

Context is another factor that influences the implementation and embedding of AMS interventions.^45–47^ In addition to understanding and targeting behaviours to improve antibiotic prescribing there needs to be an analysis of the context and organizational factors that may encourage or impede the embedding of AMS interventions.^48^ There are a variety of theories, models and frameworks from the behavioural and social sciences, such as the BCW, Consolidated Framework for Implementation Research (CFIR), the Theoretical Domains Framework and the Normalization Process Theory (NPT) that can provide a structure to understanding the determinants of the behaviours of prescribers and other HCPs along the antibiotic prescribing pathway within a particular context. These theories, models and frameworks can also guide tailor-made intervention development within a specific context.^18^^,^^49–53^ These tools are gaining utility in AMS-focused studies to understand the barriers and enablers to AMS and potentially guide theoretically informed intervention design.^54–56^ However, many of these studies have been conducted in the community or a long-term care setting.^54–56^ The studies included in this review failed to investigate the context within which interventions were implemented and did not provide any understanding of how these settings may have influenced uptake of interventions. It is important to further employ these aforementioned theories, models and frameworks in the design of AMS interventions, particularly in the acute care hospital setting where there is a greater burden of IV antibiotic use.^1^ A better understanding of context can help tailor interventions to increase likelihood of successful implementation in a range of contexts.^49^^,^^53^

In addition, most studies in this review were conducted in HICs, which have distinctly different healthcare systems from low-and middle-income countries (LMICs). LMICs have a greater burden of inappropriate antibiotic consumption and weakened healthcare systems that worsen the impact of infectious diseases and AMR.^57^^,^^58^ Hence the findings from this review are not generalizable to all countries with different income levels and contexts.

### Reporting of intervention content

Antibiotic review, in addition to other components of the antibiotic prescribing pathway,^11^ especially IVOST and optimization of DOT (which are the focus of this systematic review), involve complex behaviours that are influenced by various interacting factors such as hierarchy within the healthcare settings, the level of training of the prescriber, their knowledge and attitude to prescribing and antibiotic use and the influence of their peers and colleagues.^11^^,^^45–47^^,^^59–61^ Hence, interventions to improve antibiotic prescribing, including antibiotic review, need to include an understanding of key antibiotic prescribing and antibiotic treatment behaviours, and how they may be influenced to improve the appropriateness of antibiotic prescribing. Mapping the interventions in this systematic review to aspects of the BCW allowed a closer look at how the reported interventions may have influenced the behaviours of the targets of the interventions.

The reporting of intervention design was relatively poor in the studies included, as highlighted previously. Where multicomponent interventions had been applied, it was difficult to code them into the EPOC subcategories^17^ and the BCW^18^ intervention functions because sufficient information on the interventions was not provided. Also, the sequence of intervention implementation was unclear in some studies, as well as which specific strategies led to their reported effectiveness. Linking intervention strategies to theoretical constructs during intervention design will aid evaluation of mechanisms of action and change.^18^

Additionally, clear-cut descriptions of the mode of delivery of interventions were not provided, although for studies where interventions included delivery of educational sessions it was mentioned whether they were delivered in a group setting or individually, e.g. on clinical wards. The challenge with not being able to discern the detail of the interventions in these studies is that it would be difficult to replicate their effectiveness in other settings. Hoffman *et al.*^62^ have suggested that employing a tool such as the Template for Intervention Description and Replication (TIDieR) checklist could improve the reporting of interventions. The TIDieR checklist^62^ is useful when trying to achieve a complete description of interventions in published work. This makes it easier for other researchers and clinicians to re-implement interventions that have been shown to be effective. It also increases the chances of intervention fidelity (i.e. the degree to which an intervention is implemented as prescribed, e.g. in a protocol^63^), although this needs to be balanced with intervention adaptation (the degree to which an intervention is modified during implementation to fit local needs^64^), to ensure tailoring of interventions to different contexts. The TIDieR checklist^62^ has also been designed to include considerations of context.^65^ The minor drawback with using the TIDieR checklist is that it may not be effective enough in achieving full reporting of applied interventions such as quality improvement interventions to improve antibiotic prescribing, because it does not provide room to go into the details of the specific intended behaviours of identified actors, i.e. both the interventionists and groups targeted by the intervention.

Another way of detailing aspects of an intervention for easy replication is the ‘Action, Actor, Context, Target, Time’ (AACTT) framework.^66^^,^^67^ The AACTT framework is useful for specifying the intended behaviours (action) targeted by an intervention and identifying the key actors (HCPs in this case) that will be involved in delivering an intervention to a specified target within a specific time period in a particular context.^66^ The AACTT framework also helps to clarify the roles of different stakeholders involved in intervention delivery. It allows for better replication of interventions as it provides a clear report of components of the intervention. This framework can be applied to interventions targeted at improving the review of antibiotic prescriptions, as different parts of the antibiotic prescribing pathway involve complex behaviours across different categories of HCPs.^11^^,^^45^^,^^59^

### Long-term effectiveness of interventions

The reported interventions in the individual studies were mostly effective within the settings studied, although long-term effectiveness was only investigated in two studies.^24^^,^^30^ Long-term sustainability of healthcare interventions is important to ensure they are embedded within healthcare settings.^68^ Ideally more than 1 year is needed to allow for the dissemination of feedback on intervention effectiveness, alongside monitoring of intervention impact.^38^ Using frameworks such as CFIR and NPT can guide researchers to where AMS interventions should be focused to ensure long-term sustainability.^52^^,^^53^^,^^68^ Considering long-term sustainability and associated benefits in AMS intervention design and evaluation will provide users (e.g. HCPs, hospital management) of these interventions, and policymakers, with confidence in their effectiveness and support increased funding.

### Implications for practice

Interventions that were reported to lead to positive outcomes with respect to the review of antibiotic therapy (and subsequently IVOST and improved DOT) employed common intervention strategies. However, the poor reporting of intervention content and mode of delivery in these studies limits solid recommendations about which specific individual strategies or combination of strategies should be applied in clinical practice and how, as it is not clear which intervention strategies are more effective than others, or which intervention strategies lead to more effective implementation in the long term.

More research that focuses on the clear design of interventions with a theoretical underpinning and with investigations of context is needed in order to be able to arrive at clear recommendations for the implementation of individual intervention strategies in practice.

### Strengths and limitations

This review was conducted using a transparent systematic process based on a previously registered protocol.^14^ Transparency and rigour were achieved by following the PRISMA guidelines for conducting and reporting systematic reviews.^13^ Two to three researchers were involved in all stages of the review process ensuring that there was double-checking of decisions made by the first researcher as well as an opportunity for each researcher to make independent decisions and come to a final consensus based on discussion and agreement. This systematic review also involved a comprehensive literature search that was preceded by an extensive systematic scoping review of the current evidence for the effectiveness of all AMS interventions. This is in line with established guidance confirming that a scoping review is usually followed by a systematic review, with refined and specific aims and objectives developed based on the findings of the scoping review.^69^

As a result of the heterogeneity of study designs of the included studies, timing of intervention strategies targeted at improving review of antimicrobial therapy, and the outcomes measured, it was not possible to do a meta-analysis of the results of all studies. Also, the multicomponent aspects of the interventions and their poor reporting limited clear categorizations of these interventions. However, this was mitigated by employing the EPOC taxonomy^17^ for categorizing health system interventions based on common conceptual similarities. Although the EPOC taxonomy^17^ enabled better categorization of the reported interventions in this review, its utility was limited by its insufficient detail on the link between intervention strategies and their component parts that could be effective in achieving the desired behaviours (in this case, timely review of IV antibiotic therapy, IVOST and appropriate DOT). Using the BCW^18^ to further code the interventions allowed identification of intervention functions intended to achieve the desired behaviour.

Additionally, as the majority of intervention strategies reported were found to lead to improvements in the review of antibiotic therapy, there is the potential for publication bias.^70^ This publication bias could arise because studies with negative results are less likely to be reported in the literature and/or less likely to be accepted for publication.^70^ Hence, it is possible that more unpublished evidence may exist on the ineffectiveness of certain intervention strategies in improving the review of antibiotic therapy.

With respect to the systematic search and approach employed in this study, one key limitation is the non-retrieval of studies that were not published in English, possibly leading to reporting bias. This means that there may be studies that have targeted improved review of antibiotics that have not been included.

### Conclusions

Interventions to improve the review of antibiotic therapy in acute care hospitals have been shown to be effective in the short and medium term in the achievement of the timely review of IV and oral antibiotic therapy, IVOST and the optimization of the duration of antibiotic therapy. However, concrete recommendations on which intervention strategies are most effective compared with others cannot be made based on the findings of this review, due to limits in methodological quality of the included studies and particularly the inability to make conclusions on the direct causal linkage between interventions and their reported effectiveness.

The current evidence base lacks clear reporting of the detail of these interventions and how they were delivered. Also, the theoretical basis to the design and implementation of these interventions has not been investigated in the existing literature. This absence of theory could be expected as there has only been a period of 2 years from the publishing of the Cochrane review by Davey *et al.*^12^ and this systematic review search, which is not sufficient time for researchers to have taken on board the recommendations by Davey *et al.*^12^ to consider theory application in intervention design. Future research should focus on the design of AMS interventions with a theoretical underpinning, full specification of the components of interventions and an investigation into the effectiveness of implementing AMS interventions in different settings over longer periods of time.

## Supplementary Material

dlaa065_Supplementary_Data
