# Supplementary material for: Interventions to improve the review of antibiotic therapy in acute care hospitals: a systematic review and narrative synthesis
Source: JAC Antimicrob Resist. 2020 Sep 17;2(3):dlaa065. doi: 10.1093/jacamr/dlaa065 (PMC8210161; doi:10.1093/jacamr/dlaa065)
Supplement: dlaa065_Supplementary_Data [file dlaa065_supplementary_data.docx]

# Supplementary data

**Table S1** Search strategy and search results in MEDLINE database

| Serial No | Search term | Number of hits |
| --- | --- | --- |
| S1 | ""healthcare professional*"" | 41255 |
| S2 | ""health care professional*"" | 147094 |
| S3 | "doctor*" | 139193 |
| S4 | "physician*" | 569180 |
| S5 | "pharmacy" | 377602 |
| S6 | "pharmacist*" | 34331 |
| S7 | nurs* | 798451 |
| S8 | clinician* | 207923 |
| S9 | S1 OR S2 OR S3 OR S4 OR S5 OR S6 OR S7 OR S8 | 2002804 |
| S10 | (MH "Pharmacy") OR (MH "Pharmacy Service, Hospital") | 22383 |
| S11 | (MH "Pharmacists") | 15016 |
| S12 | (MH "Nurse Clinicians") OR (MH "Nurse Specialists") OR (MH "Nurse Practitioners") | 23838 |
| S13 | (MH "Nursing") | 50764 |
| S14 | S10 OR S11 OR S12 OR S13 | 108294 |
| S15 | S9 OR S14 | 2002804 |
| S16 | ""antimicrobial stewardship"" | 3360 |
| S17 | ""antibiotic stewardship"" | 3004 |
| S18 | ""antimicrobial prescribing"" | 2342 |
| S19 | ""antibiotic prescribing"" | 5456 |
| S20 | S16 OR S17 OR S18 OR S19 | 9734 |
| S21 | (MH "Antimicrobial Stewardship") | 536 |
| S22 | S20 OR S21 | 9734 |
| S23 | TI ( ("antimicrobial stewardship" OR "antibiotic stewardship") N5 intervention* ) OR AB ( ("antimicrobial stewardship" OR "antibiotic stewardship") N5 intervention* ) | 333 |
| S24 | TI ( ("antimicrobial prescribing" OR "antibiotic prescribing") N5 intervention* ) OR AB ( ("antimicrobial prescribing" OR "antibiotic prescribing") N5 intervention* ) | 224 |
| S25 | TI ( (antimicrobial OR antibiotic) N5 intervention* ) OR AB ( (antimicrobial OR antibiotic) N5 intervention* ) | 3824 |
| S26 | TI ( (antimicrobial OR antibiotic) N5 guideline* ) OR AB ( (antimicrobial OR antibiotic) N5 guideline* ) | 3293 |
| S27 | TI ( (antimicrobial OR antibiotic) N5 (policy OR policies) ) OR AB ( (antimicrobial OR antibiotic) N5 (policy OR policies) ) | 1729 |
| S28 | TI ( (antimicrobial OR antibiotic) N5 implement* ) OR AB ( (antimicrobial OR antibiotic) N5 implement* ) | 1577 |
| S29 | TI ( (antimicrobial OR antibiotic) N5 "audit and feedback" ) OR AB ( (antimicrobial OR antibiotic) N5 "audit and feedback" ) | 67 |
| S30 | TI ( (antimicrobial OR antibiotic) N5 program* ) OR AB ( (antimicrobial OR antibiotic) N5 program* ) | 3255 |
| S31 | TI ( (antimicrobial OR antibiotic) N5 "quality improvement" ) OR AB ( (antimicrobial OR antibiotic) N5 "quality improvement" ) | 106 |
| S32 | S23 OR S24 OR S25 OR S26 OR S27 OR S28 OR S29 OR S30 OR S31 | 11962 |
| S33 | S22 OR S32 | 18404 |
| S34 | "hospital*" | 4616716 |
| S35 | ""acute care"" | 160409 |
| S36 | ""acute-care"" | 22322 |
| S37 | "inpatient*" | 105237 |
| S38 | S34 OR S35 OR S36 OR S37 | 4706254 |
| S39 | S15 AND S33 AND S38 | 4769 |
| S40 | S15 AND S33 AND S38  Limiters - Date of Publication: 20150101-20190831; English Language | 2187 |

**Table S2:** Characteristics of the 14 included studies

| Authors (year and country) | Study aim/ objective | Methods | Participants | Intervention(s) | Intervention strategy (EPOC Subcategory^17^) | Intervention function^18^ | Theoretical underpinning |
| --- | --- | --- | --- | --- | --- | --- | --- |
| Beeler et al (2015)^23^  Switzerland | To encourage timely IV to oral antibiotic switch using computerised clinical decision support (CDS) to lead to the reduction of the duration of IV antimicrobial therapy | Prospective controlled, before-and-after trial  Time period: 2 years | *HCPs targeted*: Physicians  *Sample size*: 24599 IV antimicrobial prescriptions (6410 IV-oral switches).  *Setting*: Single site; 29 inpatient units in 1 University Hospital | *Type of intervention*: Computerised reminders in the electronic health record, for patients eligible for IV-oral antimicrobial switch  *Deliverers of intervention*: Not mentioned  *Comparison*: pre-intervention (baseline) outcomes and control group  *Outcome measures*: duration of IV therapy before switch; number of IV-oral antimicrobial switches within 60-300 hours | Reminders;  Clinical practice guidelines | Enablement; Education; Environmental restructuring | None |
| Berrevoets et al (2017) ^24^  The Netherlands | To evaluate the effect of a combined intervention targeting different barriers that hamper IV to oral antibiotic switch | Controlled before and after interventional study  Time period: 26 months | *HCPs targeted*: Physicians  *Sample size*: 1519 patients on the intervention wards and 4366 patients on the control wards  *Setting*: Single site; internal medicine wards in 1 teaching hospital | *Types of intervention*:  Intervention 1: Computerised reminders sent on day 3 of treatment, which identified patients eligible for iv-oral switch therapy.  Intervention 2: An educational program, during which each physician was given a set of pocket cards with the switch protocol. A weekly short meeting was done to optimise adherence with the protocol. During the meeting, direct feedback and education were given to the physicians covering the intervention wards.  *Deliverers of interventions*: Multi-disciplinary AMS team made up of an ID specialist, microbiologist and clinical pharmacist  *Comparison*: pre-intervention outcomes and control group  *Outcome measures*: change in the % of IV treatment exceeding >72 hours; % of patients appropriately switched to oral therapy on day 3 of treatment; change in median treatment duration. | Audit and feedback; Clinical practice guidelines;  Educational materials; Educational meetings; Reminders | Enablement; Education; Environmental restructuring; Persuasion | None |
| Eljaaly et al (2018)^25^  USA | To compare restricted antibiotic utilization before and after implementation of an AMS intervention that requires ire- authorization on day 3 of therapy | Retrospective, before and after study  Time period: 6 months | *HCPs targeted*: Physicians  *Sample size*: 83 patients in the pre-intervention period, and 83 patients in the post-intervention period  *Setting*: Single site; 1 academic community medical centre | *Types of intervention*:  Intervention 1: Initial authorization plus re-authorization if restricted antibiotics were administered for ≥3 days.  Intervention 2: Discussions between members of the AMS team and prescribing physicians about inappropriate antibiotic prescriptions  *Deliverers of interventions*: AMS team- one ID consultant, one ID pharmacist, one pharmacy practice resident and one pharmacy student  *Comparison*: initial authorization of antibiotics alone; pre-intervention outcomes  *Outcome measures*: DOT of restricted antibiotic use per patient as well as per antibiotic; LOS; hospital mortality; proportions of patients on antibiotic therapy for >4 days | Educational outreach visits, or academic detailing; Local consensus process | Environmental restructuring; Restriction; Persuasion | None |
| Foolad et al (2018)^26^  USA | To assess the impact of a multicentre, multifaceted stewardship initiative to promote compliance with Infectious Disease Society of America (IDSA) and American Thoracic Society (ATS) Community Acquired Pneumonia (CAP) recommendations regarding DOT in hospitalized CAP patients and its impact on patient outcomes. | Pre-post quasi-experimental study  Time period: 12 months | *HCPs targeted*: Pharmacists and Prescribers (including interns, residents, advanced practice providers and attending physicians)  *Sample size*: 600 patients (307 in the historical control group and 293 in the stewardship intervention group)  *Setting*: Multisite; 3 large academic medical centres | *Types of intervention*:  Intervention 1: Updated guidelines on empirical CAP treatment  Intervention 2: Distribution of pocket cards highlighting guideline updates, as well as making guidelines and pocket cards available on the hospitals’ intranet sites  Intervention 3: Educational sessions on management of CAP, guideline updates and AMS programme  Intervention 4: Prospective audit with feedback and intervention, that involved the AMS pharmacists contacting prescribers and making direct verbal recommendations on patients’ appropriate DOT.  *Deliverers of interventions*: multidisciplinary AMS team; ID Pharmacists and AMS pharmacists  *Comparison*: control group; pre-intervention outcomes  *Outcome measures*: CAP antimicrobial DOT; mortality, readmission, incidence of CDI at 30 days post-encounter discharge; compliance with IDSA/ATS DOT recommendations | Audit and feedback; Clinical practice guidelines; Educational materials;  Educational meetings | Enablement; Environmental restructuring; Education; Persuasion | None |
| Hobday et al (2018)^27^  UK | To improve the percentage completion of the 48-hour antimicrobial review box section of the inpatient drug charts to over 90% | Pre-post study employing Quality improvement (QI) methodology  Time period: 7 weeks | *HCPs targeted*: Doctors, consultants in the respiratory team, pharmacists  *Sample size*: 420 prescription charts  *Setting*: single site; 2 respiratory wards in 1 University Hospital | *Types of intervention*:  Intervention 1: Incident report email (although not initiated by the QI team) requiring pharmacists to submit an incident report when a 48-hour review was not completed.  Intervention 2: Educational talk by a consultant  Intervention 3: Including the 48-hour review status on the daily handover sheet as a reminder  Intervention 4: Verbal communication between pharmacists and doctors regarding which patients had incomplete 48-hour reviews.  Intervention 5: Visual prompts in the form of A4 posters in the doctors' consultation areas  Intervention 6: Magnets on the patient board to highlight which patients required a 48-hour review (*not implemented*)  Intervention 7: Emailing of consultants to detail improvements achieved by the 6th week of the project and encouraging continuous engagement with 48-hour review completion.  *Deliverers of interventions*: Multidisciplinary QI project team: six trainee doctors, one pharmacist, one occupational therapist, one quality improvement Darzi fellow and one respiratory consultant who acted as mentor  *Comparison*: no comparison  *Outcome measures*: Completion of the 48-hour antimicrobial review tick box on inpatient drug charts; total number of days on IV antibiotics; process measures- signing and dating of the 48-hour review section on the medicine chart; balancing measures- documentation of indication and duration of antibiotics; antibiotic indication; antibiotic type; number of days on antibiotic | Educational materials; Educational meetings; Reminder,  Educational outreach visits, or academic detailing;  Audit and feedback; Continuous quality improvement; Local opinion leaders; Clinical incident reporting | Education; Enablement; Persuasion; Environmental restructuring; Coercion | None |
| Lesprit et al (2015)^28^  France | To assess whether an AMS intervention would improve appropriateness of antibiotic use, as assessed by a blinded adjudication committee. | Prospective, randomised, parallel-group, open-label randomised controlled trial  Time period: 12 months | *HCPs targeted*: Prescribing Physician/ ward physician  *Sample size*: 246 patients  *Setting*: Multisite; 2 surgical or medical wards per hospital, in 4 University-affiliated Hospitals | *Type of intervention*: review of patients’ antibiotic therapy on day 1 and days 3 or 4 after the first prescription of antibiotic therapy, followed by verbal communication of recommendations to the prescribing/ward physician, and writing these recommendations in the medical chart  (Antibiotic guidelines were reviewed and standardized, and given to participating ward physicians before study commencement)  *Deliverers of intervention*: ID physicians  *Comparison*: usual care  *Outcome measures*: overall appropriateness of antibiotic therapy; appropriateness of antibiotic therapy at days 1, 3 and 4; DOT; improvement in clinical status on day 3 and at discharge; mortality and LOS | Educational materials; Educational outreach visits, or academic detailing; Clinical practice guidelines | Persuasion; Enablement; Education | None |
| Liew et al (2015)^29^  Singapore | To evaluate what impact early review of antibiotic prescriptions (within 48 hours of initiation of therapy) has on patient outcomes and safety. | Retrospective cohort study  Time period: 12 months | *HCPs targeted*: Physicians  *Sample size*: 5797 admissions  *Setting*: Single site; 1 acute tertiary care hospital | *Type of intervention*: Review of antibiotic prescriptions by the AMS team on the 2nd (within 48 h), 4th (giving a 72-h period for bacterial cultures to be processed) and 7th days (if applicable) of antibiotic prescription for appropriateness of DOT, followed by recommended changes in therapy discussed in a meeting.  Intervention subdivisions:  Sub-intervention 1: interventions based on empirical therapy Sub-intervention 2: Intervention based on ‘culture-directed’ therapy.  *Deliverers of interventions*: AMS team made up of an ID physician, a clinical microbiologist and clinical ID pharmacists  *Comparison*: accepted interventions versus rejected interventions  *Outcome measures*: Absence or presence of: (i) 30-day all-cause mortality (from date of audit); (ii) 30-day infection-related mortality (from date of audit); (iii) 30-day re-admission (from date of discharge); (iv) 30-day infection-related re-admission (from date of discharge); (v) 14-day CDI (from date of intervention); (vi) 14- day re-infection (from date of audit); and (vii) LOS from date of audit) | Educational outreach visits, or academic detailing. | Persuasion; Environmental restructuring | None |
| Loo et al (2019)^30^  Singapore | To evaluate the safety of and cost savings associated with early (within 24 hours of prescribing) review of prescriptions for empiric antibiotics | Retrospective cohort study  Time period: 5 years | *HCPs targeted*: Physicians  *Sample size*: 794 cases  *Setting*: Single site; 1 acute tertiary care hospital | *Types of intervention*:  Intervention 1: Development of antibiotic guidelines for empirical treatment of common infections and uploading on the hospital intranet site  Intervention 2: Two-stage prospective audit, with immediate and concurrent feedback that involved, assessing the appropriateness of prescribed antibiotics against the guidelines within 24 hours, and making recommendations to modify therapy or adjust dosing.  *Deliverers of intervention*: An AMS team made up of an ID physician and ID clinical pharmacists and AMS pharmacists  *Comparison*: accepted interventions versus rejected interventions  *Outcome measures:* DOT; post interventional length of stay (PLOS); 14-day infection-related readmissions; 14-day all- cause mortality; cost savings associated with any reduction in LOS and DOT observed. | Clinical practice guidelines; Audit and feedback | Persuasion; Enablement; Environmental restructuring | None |
| Niwa et al (2016)^31^  Japan | To determine the efficacy of an antimicrobial intervention implemented within 24 h after initiating therapy, to ensure the appropriate use of antimicrobials and to evaluate its influence on the clinical outcomes of patients with bloodstream infections | Prospective cohort study  24 months | *HCPs targeted*: Physicians  *Sample size*: 396 patients  *Setting*: Single site; 1 University hospital that provides tertiary care | *Type of intervention*: The AMS team reviewed inpatient prescriptions for IV antibiotic daily (within 24 hours of initiating therapy), employing the prospective audit (review) with intervention and feedback strategy for inappropriate prescriptions.  *Deliverers of intervention*: ID Physician and ID Pharmacist  *Comparison*: no intervention  *Outcome measures*: The number of days until administration of effective IV antimicrobials; rates of de-escalation; rate of 30-day mortality associated with bloodstream infection; 30-day reinfection rate. | Audit and feedback; Local opinion leader | Enablement; Environmental restructuring; Persuasion | None |
| Park et al (2017)^32^  Korea | To investigate physicians’ adherence to an intervention (to facilitate the conversion from IV to oral fluoroquinolones) by the AMS team and to evaluate the effect of the intervention. | Retrospective cohort study  Time period: 4 months | *HCPs targeted*: Physicians  *Sample size*: 129 cases of antibiotic courses  *Setting*: Single site; 1 University hospital | *Type of intervention*: AMS pharmacist review of antibiotic therapy in inpatient electronic medical charts and placing of notes with recommendations electronically. Additional validation of recommendations by ID physicians with further recommendations made and co-signature.  *Deliverers of intervention*: Pharmacists and ID physicians  *Comparison*: physician adherence to intervention versus physician non-adherence to intervention  *Outcome measures*: Physician's adherence to the intervention (adherence was defined as, if antimicrobial therapy was discontinued or switched to oral within 3 days of the intervention); DOT; LOS; medication cost | Educational outreach visits, or academic detailing; Local opinion leader | Persuasion;  Environmental restructuring | None |
| Riain et al (2017)^33^  Ireland | To develop a stewardship initiative focused on meropenem de-escalation to address observed doubled meropenem use | Prospective cohort study  Time period: 4 weeks | *HCPs targeted*: Physicians/Prescribers  *Sample size*: 33 patients (who were identified as receiving meropenem and were reviewed)  *Setting*: Single site; 1 University Teaching Hospital | *Type of intervention*: Review of meropenem prescriptions within 48-72 hours from the commencement of therapy or as soon as it was brought to the attention of the stewardship team, followed by a note/recommendation in the patients' charts (stop, de-escalate or continue meropenem.  *Deliverers of intervention*: Ward pharmacist; Consultant microbiologist; antimicrobial pharmacist  *Comparison*: de-escalation implemented versus de-escalation not recommended or recommended but not implemented  *Outcome measures*: Days of meropenem use and crude mortality | Educational outreach visits, or academic detailing;  Clinical practice guidelines | Persuasion; Environmental restructuring | None |
| Rizan et al (2017)^34^  UK | To determine the adherence of general surgeons to the use of local IV-oral antibiotic guidelines and to evaluate the uptake of an IV-PO prompt sheet following education | Prospective cohort study  Time period: 12 months | *HCPs targeted*: Junior general surgical doctors  *Sample size*: 200 patients  *Setting*: Single site; the general surgical ward of 1 local acute teaching hospital | *Types of intervention*:  Intervention 1: Promotion of local-hospital IV-oral switch guidelines  Intervention 2: Promotion of the results of a pre-awareness intervention programme  Intervention 3: Introduction of an IV-oral antibiotic prompt sheet designed to be used for all general surgical patients on IV antibiotics, to prompt the review of 5 guideline criteria at 48 hours and also to remind prescribers to take the patients' blood samples on day 0 and day 2. The IV-oral prompt sheet was made available electronically through emails, on shared computer drives, and hard copies were placed in easily noticeable places around the general surgical ward. Promotion was done through the use of email, posters and face-to-face presentations at meetings  *Deliverers of intervention*: Not mentioned  *Comparison*: pre-intervention outcomes  *Outcome measures*: Concordance with IV antibiotic guideline criteria at 48 hours, and if so, if IV antibiotics were switched to oral antibiotics at either 48 hours or at 48-72h; if inflammatory blood markers had been taken on day 0 & 2; documentation of the intention to review antibiotics, in the patient notes; uptake of the IV-oral antibiotic prompt sheet in the post-intervention period. | Audit and feedback; Educational materials; Educational meetings; Local consensus process; Reminders; Clinical practice guidelines | Education; Enablement; Environmental restructuring; Persuasion | Roger’s Diffusion of Innovation Model |
| Sze & Kong (2018)^35^  Malaysia | To evaluate the impact of printed AMS recommendations on early IV-PO antibiotics switch | Before and after interventional study  Time period: 4 months | *HCPs targeted*: Doctors  *Sample size*: Pre-intervention phase: 79 courses of antibiotics from 72 patients; Post-intervention phase: 77 courses of antibiotics from 76 patients  *Setting*: Multisite; 8 district hospitals | *Types of intervention*:  Intervention 1: Attachment of a printed checklist (a clinical intervention form) containing information on IV-PO switch criteria to patients’ medical notes on the day patients were eligible for the switch, with the requirement that doctors document whether they accepted or rejected the switch recommendation (with reasons)  Intervention 2: Placement of an IV-PO switch sticker by the antibiotic prescription to serve as a reminder to prescribers.  Intervention 3: One week before the beginning of the post-intervention phase, all doctors in the study hospitals received a written formal letter on the availability of IV-PO switch protocols in the wards. The protocols were attached to the letters.  *Deliverers of intervention*: Ward pharmacists  *Comparison*: usual care alone (clinical pharmacists reviewing drug charts and verbally informing prescribers on the day the patient was eligible for IVOST): pre-intervention outcomes  *Outcome measures*: Timeliness of IV to PO switch (measured in days); duration of IV antibiotics; mean LOS; antibiotic cost savings | Reminders; Educational materials; Clinical practice guidelines | Enablement; Environmental restructuring; Education | None |
| Thompson et al (2015)^36^  Canada | To determine if antibiotic prescribing patterns improve after the implementation of an IV to PO conversion clinical intervention form | Pre-post study using QI methodology  Time period: 5 weeks | *HCPs targeted*: Physicians  *Sample size*: 9 patients (eligible for IV-PO switch)  *Setting*: Single site; the oncology, intensive care, progressive care, palliative and six general internal medicine wards in 1 general hospital | *Types of intervention*:  A clinical intervention form (CIF) to prompt IV-to-PO switch (escalation of antibiotic therapy from IV fluoroquinolone to oral fluoroquinolone within 48 to 72 hours of initiating therapy) delivered over 5 plan-do-study-act (PDSA) cycles:  PDSA cycle (Intervention) 1: development of the CIF  PDSA cycle (Intervention) 2: Assessment and feedback on the CIF  PDSA cycle (Intervention) 3: piloting of the CIF   PDSA cycle (Intervention) 4: delivery of an educational presentation to hospital staff, including hospital management and clinical staff  PDSA cycle (Intervention) 5: Attaching the CIF to the charts of patients who were eligible for IV-to-PO switch.  *Deliverers of intervention*: Clinical pharmacists  *Comparison*: compliant group versus non-compliant group  *Outcome measures*: length of total antibiotic, IV antibiotic and oral antibiotic therapy; IV:PO therapy ratio; physician compliance with the CIF | Educational meetings; Local consensus process | Enablement; Education; Environmental restructuring | None |

**Table S3a:** EPOC Taxonomy^17^ definitions for interventions targeted at healthcare workers

| **EPOC Taxonomy subcategory** | **EPOC Taxonomy subcategory definition or *other definition*** |
| --- | --- |
| Audit and feedback | A summary of health workers’ performance over a specified period of time, given to them in a written, electronic or verbal format. The summary may include recommendations for clinical action. |
| Clinical incident reporting | System for reporting critical incidents |
| Clinical Practice Guidelines | Clinical guidelines are systematically developed statements to assist healthcare providers and patients to decide on appropriate health care for specific clinical circumstances'(US IOM). |
| Continuous quality improvement | An iterative process to review and improve care that includes involvement of healthcare teams, analysis of a process or system, a structured process improvement method or problem solving approach, and use of data analysis to assess changes |
| Educational materials | Distribution to individuals, or groups, of educational materials to support clinical care, i.e., any intervention in which knowledge is distributed. For example this may be facilitated by the internet, learning critical appraisal skills; skills for electronic retrieval of information, diagnostic formulation; question formulation |
| Educational meetings | Courses, workshops, conferences or other educational meetings |
| Educational outreach visits, or academic detailing | Personal visits by a trained person to health workers in their own settings, to provide information with the aim of changing practice.  *To further explain, this also included intervention strategies that involved the interventionists reviewing antibiotic therapy and providing recommendations to improve antibiotic prescribing either through verbal or written communications or recommendations to the targets of the intervention strategy.* |
| Local consensus processes | Formal or informal local consensus processes, for example agreeing a clinical protocol to manage a patient group, adapting a guideline for a local health system or promoting the implementation of guidelines. |
| Local opinion leaders | The identification and use of identifiable local opinion leaders to promote good clinical practice. |
| Reminders | Manual or computerised interventions that prompt health workers to perform an action during a consultation with a patient, for example computer decision support systems |

**Table S3b:** BCW^18^ Intervention function definitions

| **Intervention function** | **Intervention function definition** |
| --- | --- |
| Education | Increasing knowledge or understanding |
| Persuasion | Using communication to induce positive or negative feelings or stimulate action |
| Coercion | Creating expectation of punishment or cost |
| Enablement | Increasing means/reducing barriers to increase capability or opportunity |
| Environmental restructuring | Changing the physical or social context |
| Restrictions | Using rules to reduce the opportunity to engage in the target behaviour (or to increase the target behaviour by reducing the opportunity to engage in competing behaviours) |

**Table S4:** Quality appraisal and Risk of Bias Assessment

**Table S4a:** Assessment of methodological quality of Observational Cohort and Cross-Sectional Studies using the NHLBI Quality Assessment Tool^19^

| Criteria | Liew et al^29^ | Loo et al^30^ | Niwa et al^31^ | Park et al^32^ | Riain et al^33^ | Rizan et al^34^ |
| --- | --- | --- | --- | --- | --- | --- |
| 1. Was the research question or objective in this paper clearly stated? | Yes | Yes | Yes | Yes | NR | Yes |
| 2. Was the study population clearly specified and defined? | NR | No | Yes | Yes | No | Yes |
| 3. Was the participation rate of eligible persons at least 50%? | NR | CD | Yes | Yes | NR | Yes |
| 4. Were all the subjects selected or recruited from the same or similar populations (including the same time period)? Were inclusion and exclusion criteria for being in the study prespecified and applied uniformly to all participants? | NR | No | Yes | Yes | NR | Yes |
| 5. Was a sample size justification, power description, or variance and effect estimates provided? | No | No | No | No | No | Yes |
| 6. For the analyses in this paper, were the exposure(s) of interest measured prior to the outcome(s) being measured? | Yes | Yes | Yes | No | No | Yes |
| 7. Was the timeframe sufficient so that one could reasonably expect to see an association between exposure and outcome if it existed? | Yes | Yes | Yes | No | No | No |
| 8. For exposures that can vary in amount or level, did the study examine different levels of the exposure as related to the outcome (e.g., categories of exposure, or exposure measured as continuous variable)? | NA | NA | NA | NA | NA | NA |
| 9. Were the exposure measures (independent variables) clearly defined, valid, reliable, and implemented consistently across all study participants? | Yes | Yes | Yes | Yes | Yes | Yes |
| 10. Was the exposure(s) assessed more than once over time? | Yes | Yes | Yes | No | Yes | Yes |
| 11. Were the outcome measures (dependent variables) clearly defined, valid, reliable, and implemented consistently across all study participants? | Yes | Yes | Yes | Yes | No | Yes |
| 12. Were the outcome assessors blinded to the exposure status of participants? | NA | CD | NR | CD | NA | NR |
| 13. Was loss to follow-up after baseline 20% or less? | NA | Yes | NR | Yes | Yes | Yes |
| 14. Were key potential confounding variables measured and adjusted statistically for their impact on the relationship between exposure(s) and outcome(s)? | No | No | No | No | No | Yes |

**Table S4b:** Assessment of methodological quality of Before-After (Pre-Post) Studies With No Control Group using the NHLBI Quality Assessment Tool^19^

| Criteria | Eljaaly et al^25^ | Hobday et al^27^ | Sze & Kong^35^ | Thompson et al^36^ |
| --- | --- | --- | --- | --- |
| 1. Was the study question or objective clearly stated? | Yes | Yes | Yes | Yes |
| 2. Were eligibility/selection criteria for the study population prespecified and clearly described? | Yes | Yes | Yes | NR |
| 3. Were the participants in the study representative of those who would be eligible for the test/service/intervention in the general or clinical population of interest? | No | No | Yes | No |
| 4. Were all eligible participants that met the prespecified entry criteria enrolled? | No | No | Yes | Yes |
| 5. Was the sample size sufficiently large to provide confidence in the findings? | Yes | No | Yes | No |
| 6. Was the test/service/intervention clearly described and delivered consistently across the study population? | Yes | No | Yes | Yes |
| 7. Were the outcome measures prespecified, clearly defined, valid, reliable, and assessed consistently across all study participants? | Yes | Yes | Yes | Yes |
| 8. Were the people assessing the outcomes blinded to the participants' exposures/interventions? | NR | NA | No | NA |
| 9. Was the loss to follow-up after baseline 20% or less? Were those lost to follow-up accounted for in the analysis? | NR | NA | Yes | Yes |
| 10. Did the statistical methods examine changes in outcome measures from before to after the intervention? Were statistical tests done that provided p values for the pre-to-post changes? | Yes | No | Yes | No |
| 11. Were outcome measures of interest taken multiple times before the intervention and multiple times after the intervention (i.e., did they use an interrupted time-series design)? | NA | No | NA | No |
| 12. If the intervention was conducted at a group level (e.g., a whole hospital, a community, etc.) did the statistical analysis take into account the use of individual-level data to determine effects at the group level? | Yes | NA | Yes | NA |

**Table S4c:** Assessment of methodological quality using the JBI Critical Appraisal Checklist for Quasi-Experimental Studies (non-randomized experimental studies)^20^

| Criteria | Berrevoets et al^24^ | Foolad et al^26^ |
| --- | --- | --- |
| 1. Is it clear in the study what is the ‘cause’ and what is the ‘effect’ (i.e. there is no confusion about which variable comes first)? | Yes | Yes |
| 2. Were the participants included in any comparisons similar? | No | Yes |
| 3. Were the participants included in any comparisons receiving similar treatment/care, other than the exposure or intervention of interest? | Unclear | Yes |
| 4. Was there a control group? | Yes | Yes |
| 5. Were there multiple measurements of the outcome both pre and post the intervention/exposure? | Yes | NA |
| 6. Was follow up complete and if not, were differences between groups in terms of their follow up adequately described and analysed? | Unclear | No |
| 7. Were the outcomes of participants included in any comparisons measured in the same way? | Yes | Yes |
| 8. Were outcomes measured in a reliable way? | Yes | Yes |
| 9. Was appropriate statistical analysis used? | Yes | Yes |

**Table S4d:** Risk of Bias Assessment using Cochrane Effective Practice and Organisation of Care (EPOC) suggested 'risk of bias' criteria for RCTs and CBAs^21^

| Criteria | Beeler et al^23^ | Lesprit et al^28^ |
| --- | --- | --- |
| Random sequence generation | High risk | Low risk |
| Allocation concealment | High risk | Low risk |
| Baseline outcome measurements similar | Unclear risk | Unclear risk |
| Baseline characteristics similar | High risk | Low risk |
| Incomplete outcome data | Unclear risk | Low risk |
| Knowledge of the allocated interventions adequately prevented during the study | Unclear risk | Low risk |
| Protection against contamination | Low risk | Low risk |
| Selective outcome reporting | Low risk | Low risk |
| Other risks of bias | None | None |

**Table S5:** Outcomes of interventions to improve the review of antibiotics

| Outcomes |  | | Primary review outcomes | | | Other clinical/related outcomes reported |
| --- | --- | --- | --- | --- | --- | --- |
|  | **Timely review of antibiotic therapy** | **Appropriateness of antibiotic therapy (in accordance with guidelines)** | | **IV to oral antibiotic switch (IVOST)** | **Duration/days of therapy (DOT) overall** |  |
| Beeler et al^23^ |  |  | |  | Overall mean reduction in IV antimicrobial therapy duration by 18.1% (23.7 hours). The mean duration of IV antibiotics that were switched while displaying reminders (60-300 hours) was reduced in the intervention group (P = 0.0059). In the control group, 2374 IV antimicrobial therapies were administered for a mean duration of 4.26 ± 6.24 days during the baseline period, and 2432 courses for 4.48 ± 5.99 days in the intervention period (P = 0.096). |  |
| Berrevoets et al^24^ |  |  | | Significant reduction (19.3%, p < 0.001) in the percentage of IV antibiotic prescriptions >72 hours in the intervention group when compared to the pre-intervention period; a smaller reduction in the control group as well (6.1%, p < 0.05). A significant additional reduction of IV antibiotic usage of 13.2% (p = 0.014) was observed, in favour of the intervention group. *During the post-intervention period, a non-significant increase in the percentage of prescriptions >72 hours was observed in the intervention-group (p = 0.43) and a non-significant decrease in the control group (p = 0.46), with no significant difference between these groups.*  For all prescriptions, 116 were eligible for IVOST, of which 84 were switched appropriately (72%) and 32 (28%) were not switched (which was incorrect). 78 prescriptions were note eligible for switch, while 29 prescriptions could not be evaluated.  56 switch forms were generated and 20 of these led to correct IVOSTs and no incorrect IVOSTs, 31 were ineligible for IVOST and 5 were recorded as incorrectly not switched. | The median duration of antibiotic usage on the intervention wards was decreased by 0.8 day (4.0 to 3.2 days, p = 0.015) after the introduction of the intervention. No significant decrease was observed on the control wards or between both intervention and control wards. |  |
| Eljaaly et al^25^ |  |  | |  | For all restricted antibiotics, the median (IQR) DOT decreased from 5 (4–9) to 4 (3–5) days (P< 0.001) and the percentage of patients receiving restricted antibiotics for > 4 days reduced from 57.8% to 30.1% (P< 0.001). For only restricted broad-spectrum Gram-negative antibiotics (amikacin, aztreonam, cefepime, ertapenem, meropenem and piperacillin/tazobactam), the median (IQR) DOT decreased from 5 (3–6) to 3 (3–5) days (P<0.001) and DOT reduced for each of these antibiotics; however, the decrease was statistically significant only for cefepime [decreased from 6 (5–10.25) to 3 (3–5) days, P=0.018] and piperacillin/tazobactam [decreased from 5 (4–7) to 4(3–5 days), P=0.002]. For oral vancomycin, DOT decreased from a median (IQR) of 6.5 (6–7) to 3 (3–4.5) days | Oral vancomycin DOT decrease was not associated with fewer cases of CDI. For all restricted antibiotics, the LOS significantly reduced from a median (IQR) of 8 (5–17) to 6 (5–9) days (P=0.005), while hospital mortality decreased from 8 (9.6%) to 2 (2.4%) deaths, but this reduction was not significant (P=0.057). |
| Foolad et al^26^ |  |  | |  | The median (IQR) DOT for patients in the intervention group was significantly  lower compared with the control group; 6 (5–7) versus 9 (7–10) days, P< 0.001. The control group had a median of 3 days’ excess antibiotic treatment from the IDSA/ATS recommended duration and this was reduced significantly to 1 day in the intervention group (P< 0.001). In this way, a total of 586 days of unnecessary antibiotics were avoided over the period of the intervention. In line with IDSA/ATS DOT recommendations, 96.4% of the historical control group and 91.5% of the intervention group met the criteria for and should have received 5– 6 days of therapy for CAP treatment. Only one patient in the historical group and four patients in the intervention group should have received more than 10 days of therapy based on the IDSA/ATS guidelines. Appropriate DOT increased from 5.6% in the control group to 42% in the intervention group (P< 0.001). More patients in the intervention group received 5 days of therapy; 103 (35.2%) versus 15 (4.9%) patients, P< 0.001. In the intervention group, there was a reduction in longer durations of therapy (8– 14 days). | Readmission rates to the hospital for pneumonia were similar in the control and intervention groups (7.1% versus 3.8%). Also the incidence of visiting a clinic or emergency centre for pneumonia was similar between both groups (6.8% versus 4.4%). There was no difference in mortality at 30 days’ post-discharge (2.3% historical control versus 1% intervention group, P= 0.233). CDI was not discovered in either the historical group or the intervention group throughout their hospital stay or at 30 days post-discharge |
| Hobday et al^27^ | Baseline data showed only 68% of patients had their 48-hour antimicrobial review box ticked; this increased to 100% by the 11th measurement cycle and sustained after that.  At median baseline, 66.7% of 48-hour antimicrobial reviews were signed and 63.3% were dated. Both median values increased to 100% by measurement cycle 11 and remained at 100%. At median baseline, 93.3% had a clinical indication for antimicrobial documented on the drug chart rising to a median of 98.3% and 86.7% had the treatment duration documented; this rose to a median of 95% by cycle 13-14. |  | |  | The average time on IV antibiotics showed an associated reduction from a baseline median measurement of 2.25 days to 1.5 days by cycles 13–14 |  |
| Lesprit et al^28^ |  | 55 (44.7%) patients in the intervention group vs 35 (28.5%) patients in the control group received appropriate antibiotic therapy. *Appropriateness of antibiotic therapy at day 1 did not differ significantly between both groups.* While on days 3 and 4 ID review led to a significant increase in the appropriateness of therapy overall (based on treatment being adequately pursued or stopped, and optimal selection of the drug) (risk ratio: 2.19, 95% CI 1.29-3.71). | |  | The duration of antibiotic treatment was lower in the intervention group vs the control group (7 [3-14] days vs 10 [7-16] days. P value <0.003). | Clinical outcomes were similar between both groups; clinical improvement at day 3- p=0.53; clinical improvement at discharge for patients discharged after day 3- p=0.25; mortality- p=1; LOS- p=0.55. |
| Liew et al^29^ |  |  | |  | For interventions based on empirical treatment, a shorter mean DOT in the accepted group compared with the rejected group (3.61 ± 1.37 days vs. 6.25 ± 2.61 days; P < 0.001).  For culture-directed treatment, interventions led to a shorter mean DOT in the accepted group compared with the rejected group (P < 0.001). (2.26 ± 0.91 days vs. 5.56 ± 3.27 days; P < 0.001). | For interventions based on empirical treatment, a lower 30-day all-cause mortality rate (7% vs. 17%; P = 0.003) and lower 30-day infection-related mortality rate (2% vs. 10%; P = 0.002) were observed in the accepted group compared with the rejected group. There was no significant difference in LOS (P = 0.178), 14-day CDI rate (P = 0.227) or 30-day re-admissions (P = 1.000). For culture-directed treatment, interventions did not change the 30-day all-cause and 30-day infection-related mortality rates (P = 1.0), 30-day re-admission rates (P = 1.0), LOS (P = 0.261), 14-day re-infection rate (P = 1.0) and 14-day CDI rate (P = 0.389). |
| Loo et al^30^ |  |  | |  | Overall acceptance rate of interventions- 72.9%. There was a significant reduction in the mean duration of antibiotic use by 2.61 days (P < 0.01) in the accepted group (2.72 ± 3.04 days) compared with the rejected group (5.33 ± 2.54 days). | The PLOS was significantly shorter by 7.41 days ( P < 0.01) in the accepted group (7.98 ± 13.14 days) than in the rejected group (15.39 ± 22.62 days). The estimated cost savings for patients in the accepted group was SGD 10, 817 per patient case. Of the 579 patients in the accepted group, 18 (3.1%) died from all-cause mortality within 14 days of intervention, while 12 (5.6%) of 215 patients in the rejected group died within 14 days of intervention ( P = 0.10). Of the 731 surviving patients, 38 of 543 patients (7.0%) in the accepted group and 18 of 188 patients (9.6%) in the rejected group were readmitted for infection-related causes ( P = 0.25) |
| Niwa et al^31^ | The number of days to effective IV antimicrobial treatment from the onset of infection was significantly shorter in the intervention group (p=0.022). | On day 2 from the onset of infection, the rate of antimicrobial therapy appropriateness to the identified pathogens increased significantly in the intervention group, from 64.5% to 79.2% (p=0.001). | | There was a significant increase in the rate of de-escalation based on culture results after the intervention (55.5% non-intervention vs 81.7% intervention, p<0.001). |  | The rate of 30-day mortality associated with bloodstream infection was significantly lower in the intervention group (5.4% intervention vs 11.4% non-intervention, p=0.030). Finally, the rate of re-infection within 30 days was lower in the intervention group (2.8% intervention vs 8.2% non-intervention, p=0.063) |
| Park et al^32^ |  |  | |  | The total duration of antimicrobial administration was 7 and 11 days, respectively, in both groups (P = 0.034).  The duration of IV antimicrobial administration was 6 days for the intervention-adherent group and 10.5 days for the intervention-non-adherent group (P <0.001). | Length of hospital stay was longer (10 days vs. 14.5 days) in the intervention-non-adherent group (P = 0.004). There was a reduction in the period from the intervention to discharge in the adherent group as compared to the non-adherent group (3.0 days vs. 8.0 days; P <0.001). Costs of IV fluoroquinolones per patient were significantly lower in the adherent group than the non-adherent group (P <0.001). |
| Riain et al^33^ |  |  | |  | The median days of meropenem use in patients for whom recommendation to de-escalate was followed was 4.5 days (range 2–19) compared with 14 days (range 6–84) in those where de-escalation was not recommended or the recommendation was not implemented. (p = 0.001). | Crude mortality was 33 % in those patients de-escalated and 24 % in those not de-escalated (p > 0.05). |
| Rizan et al^34^ | 27.27% pre-intervention patients switched from IV to oral antibiotics between 48 and 72 hours met the guidelines for switching. This reduced to 8.4% of patients that met the criteria post-intervention. There was a significant increase in the percentage of patients that had a documented intention to review IV antibiotics at 48 hours, post-intervention (p<0.05). *The IV-oral antibiotic prompt sheet was not used in any of the post-intervention patients.* |  | | *No significant difference in the percentage of patients that were switched from IV to oral antibiotics at 48 hours (p=0.105),* although there was a trend towards an increase in the percentage of patients who were switched from IV to oral antibiotics between 48 and 72 hours (p=0.053). *During the pre-intervention period, 60% of patients that met the criteria for IV to oral antibiotic switch were switched to oral antibiotics within 72 hours compared to only 22.22% following the intervention*. 25% of patients met the criteria for switch prior to the intervention compared to after the intervention when this was reduced to 20%. |  | There was no difference in the number of patients that had inflammatory marker bloods taken on day 0 and day 2. |
| Sze & Kong^35^ |  |  | | IV antibiotics were switched faster in the post-intervention group (0.21 days) versus the pre-intervention group (1.83 days) (p<0.0001) | In the post-intervention group, mean duration of IV antibiotics was 2.81 days (SD=1.77), compared to the pre-intervention group where overall mean IV antibiotic duration in the ward was 4.05 days (SD=2.81) (p<0.0001) | LOS in the post- intervention group was significantly reduced by 1.44 days compared to the pre-intervention group [4.09 days (SD=1.73) vs 5.53 (SD=3.22) days, p=0.001]; median antibiotic cost savings was significantly higher in the post-intervention group [MYR 21.96 (IQR=23.23) vs MYR13.10 (IQR=53.76), (p=0.025)] |
| Thompson et al^36^ |  |  | |  | The average length of time spent on IV was 42% longer in those without physician compliance, while the IV:PO ratio was 55:1 and three out of four patients were on IV for four days or longer. IV:PO ratio was 1:1 in the compliant group, while only one of the four was on IV for four or more days. Hospital wide fluoroquinolone usage improved during the post-intervention phase (34.7% IV: 65.3% PO compared to 39.4% IV: 60.6% PO in the pre-intervention phase). |  |
